# Deep Neural Network Models for Colon Cancer Screening

**DOI:** 10.3390/cancers14153707

**Published:** 2022-07-29

**Authors:** Muthu Subash Kavitha, Prakash Gangadaran, Aurelia Jackson, Balu Alagar Venmathi Maran, Takio Kurita, Byeong-Cheol Ahn

**Affiliations:** 1 School of Information and Data Sciences, Nagasaki University, Nagasaki 852-8521, Japan; kavitha@nagasaki-u.ac.jp; 2 BK21 FOUR KNU Convergence Educational Program of Biomedical Sciences for Creative Future Talents, School of Medicine, Kyungpook National University, Daegu 41944, Korea; prakashg@knu.ac.kr; 3 Department of Nuclear Medicine, School of Medicine, Kyungpook National University, Kyungpook National University Hospital, Daegu 41944, Korea; 4 Borneo Marine Research Institute, Universiti Malaysia Sabah, Kota Kinabalu 88400, Malaysia; aureliajcksn@gmail.com (A.J.); bavmaran@ums.edu.my (B.A.V.M.); 5 Graduate School of Advanced Science and Engineering, Hiroshima University, Higashi-Hiroshima 739-8521, Japan; tkurita@hiroshima-u.ac.jp

**Keywords:** artificial intelligence, colorectal cancer, interpretation, neural network, transfer learning, transparency

## Abstract

**Simple Summary:**

Deep learning models have been shown to achieve high performance in diagnosing colon cancer compared to conventional image processing and hand-crafted machine learning methods. Hence, several studies have focused on developing hybrid learning, end-to-end, and transfer learning techniques to reduce manual interaction and for labelling the regions of interest. However, these weak learning techniques do not always provide a clear diagnosis. Therefore, it is necessary to develop a clear explainable learning method that can highlight factors and form the basis of clinical decisions. However, there has been little research carried out employing such transparent approaches. This study discussed the aforementioned models for colon cancer diagnosis.

**Abstract:**

Early detection of colorectal cancer can significantly facilitate clinicians’ decision-making and reduce their workload. This can be achieved using automatic systems with endoscopic and histological images. Recently, the success of deep learning has motivated the development of image- and video-based polyp identification and segmentation. Currently, most diagnostic colonoscopy rooms utilize artificial intelligence methods that are considered to perform well in predicting invasive cancer. Convolutional neural network-based architectures, together with image patches and preprocesses are often widely used. Furthermore, learning transfer and end-to-end learning techniques have been adopted for detection and localization tasks, which improve accuracy and reduce user dependence with limited datasets. However, explainable deep networks that provide transparency, interpretability, reliability, and fairness in clinical diagnostics are preferred. In this review, we summarize the latest advances in such models, with or without transparency, for the prediction of colorectal cancer and also address the knowledge gap in the upcoming technology.

## 1. Introduction

Colorectal cancer is the third most common cancer worldwide and was the second most common cause of cancer-related deaths in 2018 [1,2]. Endoscopic removal of precancerous lesion is considered the best way to prevent colorectal cancer. The prognosis of patients with colorectal cancer can be improved by early detection of cancerous lesions; thus, there is a need for reliable, early, and accurate endoscopic diagnosis [3,4,5,6]. Colonoscopy is the gold standard for screening colorectal lesions [7,8,9]. However, the rate of missed polyp detection during colonoscopy increases according to the expert’s knowledge of endoscopy [10,11,12]. Hence, artificial intelligence (AI) technologies could help in reducing the skill gaps among clinicians and thereby decrease the rate of missed lesions during colonoscopy [13,14,15,16].

Given their shared features, colon cancer and rectal cancer are often indicated together. In this study, rectal, colorectal, and other types of cancers related to colon cancer were analyzed using deep learning [17,18,19,20]. Convolutional neural network (CNN)-based standard deep structures have been extensively used to segment and classify colon lesions as being distinct from other unwanted regions [21,22,23,24,25]. However, to date, most of the AI for computer-aided diagnostic systems discussed in the literature have relied on extensive manual parameter setting for feature pattern extraction, which affects the outcomes [26,27,28,29]. Hand-crafted features with a feature selection module are required before implementing a neural network, which can be automatically interpreted and markedly improves the accuracy of colorectal cancer diagnosis [30,31,32].

Two separate colorectal cancer neural networks that were developed for segmentation and classification of colon glands achieved accuracy in detecting benign and malignant cancer [33]. Although these showed good performance, their frameworks typically showed poor performance in detecting lumen and gland size variations. This may be due to manual parameter settings for reducing various illumination conditions, which affects the region of interest for the classification of features. This causes bias and is undesirable for lesion detection.

Most previous systems relied on preprocessing to extract features for deep learning structures [34,35,36,37]. Only a few of these systems used end-to-end learning, allowing automatic extraction of features from images without requiring expert feature detection [38,39,40,41]. However, the information essential for clinical decision-making based on these architectures is often hidden in high-dimensional spaces and is not comprehensible to humans. It is, therefore, essential to address the interpretability and explainability of decisions in healthcare. If these aspects are not addressed, these challenges may limit the chances of adoption of an automatic system in real-world clinical practice. Thus, it is important to develop AI approaches that can generate additional new attentive information in order to gain insights into the behavior of networks. This is not yet widely available or exploited in current diagnostics. However, a few methods that have approached the interpretability of these networks have been developed [42,43]. 

Network training with unbalanced data distributions produces high-precision and low-recall predictions and is severely biased toward the majority classes [44,45]. This is unacceptable because of potential false negatives, which are more important than false positives in cancer diagnosis. This also emphasizes the importance of the development of more reliable AI techniques and interpretations. 

Although an increasing number of AI systems for the detection of colorectal cancer have been developed, they have not focused on interpretability, reliability, or the potential to design a cost-effective AI system-based diagnostic framework. Our systematic review explains the descriptions of the recent AI learning based on hybrid, end-to-end, knowledge-transferring, explainable AI and sampling methods and elucidates its advantages and disadvantages for more reliable detection. We also investigate the gaps in subsequent decision-making, identify future challenges, and present further recommendations. We have summarized literature retrieved from PubMed on the latest developments in deep learning (DL) models focusing on colorectal cancer.

## 2. Imaging Modalities

To remain consistent and avoid a selection bias towards the datasets, several studies used varied images and sizes. One study used 224 × 224 RGB images with a resolution of 256 × 256 pixels of 200 normal tissue samples and 200 tumor samples [39]. The sliding-window technique was used to break these down into smaller images. Other studies used various images, such as endoscopic and whole slide images (WSI) for the detection of colon cancer [46,47]. Another study used a larger image size (768 × 768 pixels) to preserve tissue architecture information and reduce computational cost, as opposed to a smaller patch size (384 × 384 pixels), which produced the same result but had a higher computational cost [48]. In the study using WSIs of cytokeratin immunohistochemistry obtained using a digital slide scanner, images were standardized to set 1 μm = 1 pixel and were saved as non-layered joint photographic experts group (JPEG) images, which were then converted into binary images after deletion of non-cancerous areas [49]. Another study utilized an automatic cropping approach, which removed black margins and resulted in a square image with a 1:1 ratio [50].

## 3. Methodological Approaches

**Figure 1:**
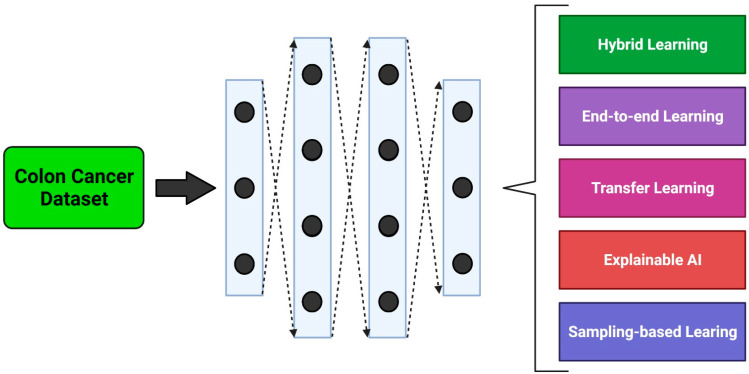
An overview of deep learning models in colon cancer detection and diagnosis. Created with BioRender.com (accessed on 1 July 2022).

Recent studies using DL models for recognizing colorectal polyps were able to achieve good performance with a large amount of data. However, the predictions of nonpolypoid lesions were unclear [34,37]. This is clinically critical, because the target task of the developed AI system is the accurate identification of nonpolypoid lesions, given that this is not a difficult task for an endoscopist. Furthermore, an AI system that can achieve superior sensitivity and specificity by preventing missed lesions, without being user-dependent, would be highly useful in clinical trials. Such a system could be particularly valuable for improving reliability and reducing interobserver variability. The DL methods explained in the following sections were originally implemented for specific tasks and can be applied to colon screening and diagnostic tasks using various types of images (Figure 1).

### 3.1. Hybrid Learning Methods

Hybrid learning methods combine various algorithms, processes, or procedures from different applications. In situations where datasets are lacking, extracting the most relevant information from the available datasets is important for analysis. This technique can be helpful, particularly for extraction and classification of colon cancer. Ghosh et al. developed a hybrid learning model that combined two machine learning techniques involving supervised (SL) and unsupervised learning techniques for the detection of colon cancer. This yielded better accuracy than existing approaches and could potentially be used for real-time cancer detection [51]. This study evaluated data clustering by K-means, the Girvan–Newman algorithm, and Mahalanobis distance-based clustering, followed by feature selection and dimensionality reduction based on principal component analysis. The data was then fed into an artificial neural network (ANN) for colon cancer classification. Another study on colorectal cancer involving small datasets utilized the CNN system ConvNet from the Visual Geometry Group (VGG) and modified it in five different ways. The configuration model that could best identify tumor images was then evaluated [39], and it was found that the best model was the one that had the most weight layers and depths and displayed the most stable accuracy and loss curves. However, that study did not include some variables, such as large images, to ease computation. To overcome gaps in colonoscopy, i.e., the detection of non-polypoid colorectal lesions, Yamada et al. first trained a Faster R-CNN model with an ImageNet dataset and then further trained it with images of polypoid lesions, consecutive lesions, and noncancerous tissues taken from videos to detect features such as edges and curves [46]. In another study, the issues associated with small datasets was addressed by a method that included images of polyps in the dataset. This produced more samples for training, while at the same time it preserved the realistic features of the images [52]. This model improved the colonic polyp detection rates and also reduced the false-negative rate. Furthermore, Ho et al. utilized a hybrid AI model using training data with annotations from pathologists. They applied a classical machine learning classifier and a Faster R-CNN model with ResNet-101 for glandular segmentation and achieved high detection and sensitivity rates for colorectal features [47]. 

The segmentation model provides detailed results based on individual samples and enables pathologists to derive further quantitative data from WSIs. For instance, the application of segmentation not only allows the study to segment colonic tissues into categories, but also segments other structures, such as blood vessels and inflammatory lesions. Yu et al. compared SL and semi-supervised learning (SSL) and showed that the latter performed better and had better generalization abilities than SL with a small amount of labeled data and large amounts of unlabeled data [53]. They also demonstrated that the SL model had reduced generalization performance when training data and testing data were not obtained from the same source. In a study by Urban et al., a different CNN model was trained with images from ImageNet, resulting in a highly accurate model with potential for real-world use [34]. Moreover, an accurate, reliable, and active (ARA) strategy was implemented in a new Bayesian DL CNN model (ARA-CNN), which was tasked with classifying colorectal tissue and which provided an estimated uncertainty using variational dropout to quicken the learning process [54]. The model, which was inspired by Microsoft ResNet and DarkNet 19, displayed high accuracy and surpassed other methods that were trained using the same dataset. Furthermore, the detection of colorectal cancer using a DL-based Inception V3 model pre-trained with an ImageNet dataset and combined with segmentation from digitized hematoxylin–eosin (H&E)-stained histology slides yielded good performance [48]. 

A computer-aided diagnostic system for endocytoscopic imaging can support non-experts in diagnosing lesions without prior training. Such a system showed an accuracy rate comparable to those of experts, and hence is more beneficial to trainees, as it only requires the push of a button to obtain a real-time diagnostic output [55]. Takamatsu et al. used image processing combined with machine learning in Image J software to generate a prediction model for colorectal cells in lymph node metastasis (LNM) and used cytokeratin immunohistochemistry obtained from a digital slide scanner for an accurate detection of cancer foci. It successfully predicted LNM [50]. A further study sought to develop a mass screening method for determining colorectal cancer risk. To this end, they evaluated seven SL models, i.e., linear discriminant analysis, support vector machine, naive Bayes, decision tree, random forest, logistic regression, and ANN. They followed this up with six imputation methods to deal with missing data (mean, Gaussian, Lorentzian, one-hot encoding, Gaussian expectation-maximization, and listwise deletion) [56]. It was found that the model combining ANN and Gaussian expectation-maximization fared best and had the potential to be used as a screening tool for early detection of colorectal cancer. Wan et al. developed the early cancer prediction algorithm model, based on an existing model (the nonnegative matrix factorization method), but with reduced matrix dimensionality and removal of repetitive data, resulting in more interpretable data [57]. 

Utilizing CNNs, such as AlexNet and Caffe, on colon endoscopy cases is useful in detecting protruding, flat, and recessed lesions. This was proven to yield accurate diagnoses and good areas under the receiver operating characteristic curve (AUC), as demonstrated by Ito et al. [58]. Tamai et al. investigated magnifying narrow-band imaging (M-NBI). This is a detailed observation approach usable in endoscopic diagnosis of colorectal lesions, albeit requiring knowledge and experience. They demonstrated its potential to be utilized with computer-aided diagnosis [59]. The software was then used to divide images of colorectal lesions into three groups: hyperplastic polyps, adenoma/adenocarcinoma lesions, and submucosal-deep lesions. Some diagnoses differed from those of expert endoscopists, which led the authors to believe that this model may have limitations in diagnosing villous lesions. 

In a study focusing on the diagnosis of colorectal adenoma, training slides were accurately labeled using a custom-developed system for annotation on iPad. The authors investigated a DL model based on DeepLab v2 with ResNet-34 and found that it yielded performance on par with that of pathologists [60]. The study also demonstrated that the deeper the network, the more information was displayed, such as gland shape, nucleus, and cell form. The report also stated that the model identified abnormalities in the glands as adenomatous glands. Rathore et al. developed a novel strategy that combined textural and geometric features of colon tissues and traditional features for the detection of colon cancer cells, and its classification into normal and malignant cases [61]. The study involved the usage of a hybrid feature space based on colon-classification; morphological, texture, scale-invariant feature transform; elliptic Fourier descriptors; and support vector machines as classifiers to extract and classify the datasets. In addition, Nadimi et al. developed a CNN by improving ZF-Net, a model that combines transfer learning, pre-processing, and data augmentations, before deploying it to a Faster R-CNN to restrict images to regions that contained colorectal polyps. This approach yielded high accuracy in autonomous detection of polyps and demonstrated high interpretability in sensitive regions by providing saliency maps [62].

### 3.2. End-to-End Learning Methods

In order to improve reliability, end-to-end DL models were considered for colon cancer identification. End-to-end (e2e) learning is the process of training a complex learning system by applying descending gradient-based learning to the system [63]. Simply put, models learn all the steps between the initial input phase and final output phase, and these parts are simultaneously trained. Some of the studies that apply e2e learning methods include the neural Turing machine, differentiable neural computer vision-based navigation in 3D environments, and value iteration networks [64,65,66,67]. While these studies have showcased successful models and techniques, quite a few noteworthy limitations remain, such as poor local optima, vanishing gradients, ill-conditioned problems, and slow convergence in different circumstances. Specifically, the development of the network architectures becomes more complex [63]. However, some of these limitations were efficiently overcome in the study by Buendgens et al., who applied e2e learning methods in a non-annotated routine database without manual labels. It accomplished good predictive performance in the identification of several diagnoses from gastrointestinal endoscopy images. This displays the potential of weakly supervised AI in clinical imaging modalities, in contrast to claims that manual annotations are a bottleneck for the future clinical application of AI [49]. The image dataset was preprocessed in MATLAB R2021a, and the ResNet-18 model was trained with the datasets. The model was capable of diagnosing inflammatory, degenerative, infectious, and neoplastic diseases from raw gastroscopy and colonoscopy images. It was able to detect the presence of diverticulosis, candidiasis, and colon and rectal cancer by learning the visual patterns of gastrointestinal (GI) pathology directly from the examination labels. In another study on the histopathological classification of gastric and colonic epithelial tumors and lesions, the authors trained CNNs and recurrent neural networks (RNNs), which included the Inception v3 network, to classify WSIs of biopsy specimens from the stomach and colon into adenocarcinoma, adenoma, and non-neoplastic tissue [68]. When tested on datasets obtained from The Cancer Genome Atlas (TCGA) with a mix of formalin-fixed paraffin-embedded (FFPE) and flash frozen tissues, the model was capable of generalized adenocarcinoma prediction, despite being largely trained on biopsies. The max-pooling aggregation method (MP-AGG) for WSI classification, demonstrated a higher log-loss than the RNN aggregation method. The probabilities of MP-AGG require a high cut-off threshold, and the method is more prone to errors in classification. Pinckaers and Litjens utilized gland segmentation datasets to train three models, i.e., a baseline U-Net model, another U-Net model with fewer filters and ordinary differential equation blocks (called U-Node), and a trained U-Node network (called U-ResNet), to predict and separate individual colon glands [69]. The U-Node network used fewer parameters compared to the other two proposed models and was able to improve segmentation. The study also showed that the neural ordinary differential equation improved the segmentation results in terms of memory load and parameter counts.

### 3.3. Transfer Learning Methods

**Figure 2:**
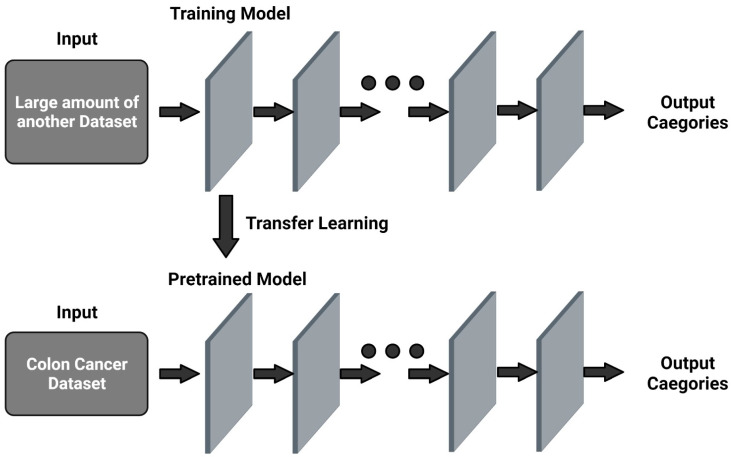
An overview of transfer learning model. Created with BioRender.com (accessed on 1 July 2022).

Transfer learning is a technique that transfers knowledge gained from a machine learning model used to address one problem to another model to solve a different but related problem. Transfer learning involves transfer of information to new tasks while entirely depending on the previously learned tasks (Figure 2). It has several main advantages over other training models, such as a better starting model, higher accuracy, and faster training [70]. There are two similar approaches, one of which involves using a pre-trained model to transfer knowledge to a target model and adapting the features of the source model, while the other involves developing a new model from scratch to transfer knowledge from its main task, and then explicitly training it with available information [71]. Transfer learning based on the AlexNet model was adopted to learn effective classification features [58]. This method increased the limited number of colon lesion images and improved the screening performance for colorectal cancers. A study that utilized the former method to study colorectal cancer using confocal laser microscopy images with various transfer learning methods from the ImageNet dataset found that this approach yielded improved performance compared to the latter method, despite functioning differently according to different models and delegated tasks [72].

In another study, a fully automated lymph node detection and segmentation method was generated through transfer learning techniques, namely by fusing T2- and diffusion-weighted images of multiparametric magnetic resonance imaging to the model Mask R-CNN to improve the performance of magnetic resonance imaging-based lymph node detection and segmentation. The model performance was evaluated based on sensitivity, positive-predictive value, false positive rate per case, and Dice similarity coefficient [32]. The model performed significantly better and faster than junior radiologists using manual detection. In addition, transfer learning methods are able to overcome issues such as a lack of rich WSI datasets. Hamida et al. used several CNNs, such as AlexNet, VGG, ResNet, DenseNet, and Inception, for studying the classification of patch-level colon cancer WSIs, of which ResNet presented the highest accuracy [73]. A pixel-wide segmentation approach for colon cancer was also applied using U-Net and SegNet models in the same study, in order to highlight regions of colon cancer in the slides. This revealed that SegNet had a higher accuracy than U-Net. Despite its exceptional performance, SegNet has a high computational cost. Additionally, Hamida et al. also compared different transfer learning strategies deployed in various CNN models and found that the models demonstrated low accuracy when learning from scratch, whereas pretraining the models resulted in better performance, although the accuracy of classification was unknown. However, fine-tuning the models produced the best performance among all the strategies and enabled rapid scanning of the datasets. Furthermore, Malik et al. investigated the usability of DL-based methods, namely a deep CNN with a limited amount of labeled data and low-resolution histology images for colorectal cancer identification and detection [74]. 

In contrast to the findings of Hamida et al., it was found that training a CNN from scratch resulted in higher accuracy and a more consistent detection rate than that of other fine-tuned models, whereas existing deep CNN models trained with transfer learning approaches produced the most superior identification of cancer. Kather et al. trained several CNNs, namely VGG19, AlexNet, SqueezeNet 1.1, GoogLeNet, and ResNet50, to identify tissue types that are abundant in histological images of colorectal cancer, including those that are non-tumorous. These were also able to decompose complex tissues into constituents and aggregated the score of the abundance of the tissue parts [75]. The VGG19 model performed best and was also able to recreate the morphological features learnt from the datasets and visualize tissue structures via the DeepDream approach. This was continuously applied to larger images and WSIs, showing a high classification accuracy that was on par with human vision. Gessert et al. utilized transfer learning approaches, including learning from scratch, partial freezing variants, and fine-tuning, in the VGG16 and Inception v3 models, with a small number of datasets [72]. The training-from-scratch method performed extremely poorly, whereas there were no significant differences between the partial freezing variants and fine-tuning strategies. The study also demonstrated that, while transfer learning improved performance, the optimal strategy differed for various models and classification tasks. 

Learning transfer with a pretrained model on ImageNet datasets, based on various CNN models for colorectal cancer using histology-stained slides, replaced the final classification layer and trained the whole network with a stochastic gradient descent with momentum. The VGG19 showed the best performance of all the networks, within an acceptable training time [75]. Another study implemented a modified VGG-based CNN model on colorectal histology images to classify normal and tumor tissue samples. This system accurately classified 294 out of 309 normal tissue images, and 667 out of 719 tumor tissue images [39]. Each of the above studies had its limitations, such as the use of a relatively small number of datasets for generalization, a weak learning procedure for an appropriate level of support for the diagnostic decision, and nontransparent high prediction accuracy in complex model architectures. It is imperative that the connection between features and predictions be comprehensible from the algorithm. Therefore, if the generated AI algorithm contributes to a clinical decision, it should be easy for clinicians to understand why a specific output was produced and how it was characterized.

### 3.4. Explainable Learning Methods

When dealing with medical data, besides demonstrating accurate prediction, it is important for models to justify uncertainties in results, as cases that are more complex should be further inspected by humans. These “failed” prediction data can then be annotated by experts and turned into a new training set [76]. This is where explainable models come into play. Explainable AI (XAI) involves processes or methods that allow users to understand the results produced by machine learning models, their impacts, and potential biases. While some models are able to compute accurate predictive data, some are not able to supply justification for their decisions. Most of the aforementioned models for colorectal cancer have used standard DL structures. Moreover, these approaches mostly focus on increasing the accuracy of the final results [68,77,78,79]. Hence, very few studies have mentioned any significant evidence that contributes to the decision outcomes [41,42,43,44,45]. Korbar et al. developed a deep ResNet visualization network for detection of colorectal polyps [42]. They established a pretrained ResNet-101 classification model with labeled patches of stained slide images. Furthermore, the classification could be projected back to the input pixel space to indicate parts of the input image that were key to the classification. This approach used a visualization model that identified regions and features of interest. A fully convolutional ResNet would be useful for visualizing the output in the last layer and to find the regions of interest for pathologists to analyze and confirm the classification of the model. Similarly, Raczkowski et al. addressed misclassified labels using an active learning-based Bayesian CNN model for classifying colorectal cancer [54]. This model was initially trained on a small dataset and on a dataset extended by using new samples, to reduce the entropy in the data analysis. 

The explainable AI system using a cumulative fuzzy class membership criterion for the classification of colorectal cancer tissues complements its decision with three types of information: visualization of the most important regions for decision, visualization of unwanted regions, and semantic explanation [43]. The use of the membership criterion proved to be a reliable and accountable explainable classifier in the decision-making process in clinical trials, with a highly satisfactory performance. In multiple instances, a fully convolutional network with attention to pooling architecture has been used to aggregate interpretable features of colorectal cancer patterns [80]. A pretrained VGG using the ImageNet dataset have been used to extract features for each image patch and adopted K-means clustering to cluster patches based on their extracted features. This was found to be more effective and suitable for huge datasets and showed better interpretability in locating important patterns and features, which contributed to accurate prediction of survival in patients with cancer.

Sabol et al. used a plain CNN model to generate an explainable Cumulative Fuzzy Class Membership Criterion (X-CFCMC) model that could be used to classify images and WSI segmentation on histopathological cancer tissues. It rationalized its decisions using three main methods: semantically explaining the possibilities of misclassification, displaying the training samples that were responsible for a certain prediction, and showing the training samples from other conflicting classes [43]. The pathologists involved in the study preferred the X-CFCMC model over the plain CNN model, as it was more useful and reliable. Another model utilizing XAI methods, namely the layer-wise relevance propagation method, was tested to identify various types of tumor entities, and it produced results that were consistent with experts’ insights and provided visual explanations in the form of heat maps [76]. These explainable heat maps assisted in detecting biases that could potentially affect the generalization abilities of the models, such as biases affecting the entire dataset, biases correlated to a specific class label by chance, and sampling biases. Moreover, Korbar et al. demonstrated the capabilities of XAI-based techniques, using gradient-based visualization approaches, for explaining reasons for classification in WSI analysis for different types of colorectal polyps, with minimal costs and easy interpretability [42]. For this study, a ResNet model was used to classify colorectal polyps on labeled patches of H&E-stained WSIs, and to justify the outcomes using a gradient-based approach. The DL architecture and imaging modality based on explainable AI and sampling studies are shown in Table 1.

### 3.5. Sampling Methods

The basic properties of AI systems should include transparency, interpretability, and reliability to provide trust and fairness in clinical diagnostics. These qualities improve discrimination ability and diminish potential mistakes. The majority of AI techniques have been designed based on balanced class distributions. However, when trained on imbalanced data, such techniques are biased toward the majority class at the expense of the minority class, degrading their overall performance [44,45,81]. Data imbalance poses a significant challenge for traditional learning algorithms. Hence, a few studies using DL methods in colon cancer have approached this data distribution problem using data-level and algorithm-level methods. Koziarski et al. utilized the oversampling technique in the image space to enhance a large amount of data to train a CNN and implemented it by sampling in the feature space to fine-tune the last layers of the network [81]. They revealed that higher levels of imbalance between classes highly affected the classification performance. Thus, they concluded that the decrease in the number of observations used for training was not only the reason for the performance decline, but that data imbalance was also an important factor. Hong et al. developed a novel algorithmic-level loss function, which combined cross-entropy with asymmetric loss in EfficentNet and U-Net models [45]. This identified each pixel individually by comparing the class predictions and producing a better balance between precision and recall for colon cancer polyp segmentation. Shapcott et al. used systematic random sampling and adaptive sampling in the CNN architecture to overcome the imbalance problem and achieved significant improvements in colorectal cancer diagnostic performance [44].

## 4. Results and Discussion

It is important to focus on what the network is learning and interpret the pixel space visualizations by an attention-based network, as these represent the regions of interest that should be located and used to confirm the classification outcomes of the model. Visualizing particular features in such deep network architectures can result in the highest probability of success in the diagnostic decision-making. Modifications to the VGG-inspired CNN model ConvNet were evaluated by identifying colorectal cells, yielding values of 93.48%, 0.4385, 95.10%, and 92.76%, for accuracy, loss, sensitivity, and specificity, respectively [39]. Ghosh et al. reported that the proposed classifiers had the highest classification accuracy (98.60%) among classifiers, ranging from 88.71% to 98.40% [51]. Furthermore, the diagnostic performance of AI and endoscopists yielded sensitivities of 97.30% and 87.40%, respectively, specificities of 99.00% and 96.40%, respectively, and processing times of 0.022 s/image and 2.4 s/image, respectively [46]. In another study, researchers achieved an accuracy exceeding 92% when using hybrid models that automatically detect colon cancer [61]. Li et al. discussed the accuracy of hybrid models that combined available features and variables to yield improved accuracy in both training and validation datasets (>90% and >85%, respectively), aside from significantly improving the prediction performance of the hybrid model [82]. In some studies, the AI model achieved an AUC of 0.917, with high sensitivity (97.4%) in detecting high-risk features of dysplasia and malignancy [47]. In another study, the labeled patches from WSIs achieved accurate patch-level recognition [83]. However, when SSL was used, only about one-tenth of labeled patches used in the SL testing and 37,800 unlabeled patches were used to achieve a similar AUC [53]. In another study, AI operating on real-time detection of polyps (1 frame in 10 ms) was able to detect the presence of polyps with an accuracy of 96.4% and an AUC of 0.991, using a CNN model that was first trained on the ImageNet data and then on an available polyp dataset [34]. Additionally, ARA-CNN was found to perform better, by 18.78%, than other models using the same dataset [54]. Another hybrid learning approach demonstrated that this technique produced a median accuracy of 99.9% for healthy tissue slides and 94.8% for cancer slides, as compared to pathologist-based diagnosis from clinical samples [48]. A hybrid learning model that was modified from ZF-Net had an accuracy of 98.0%, a sensitivity of 98.1%, and a specificity of 96.3% [62]. A study by Iizuka et al. demonstrated high AUC values (0.96–0.99) for adenomas in the gastric and colonic epithelium by applying the same techniques, with few limitations [68]. A study on weakly supervised e2e AI in gastrointestinal endoscopy found that the AUC for the diagnoses of 13 diseases had a value of 0.7–0.8 and was able to predict the presence of colorectal cancer with an AUC > 0.76 [49]. The accuracy of a few CNN models in recognizing features in colorectal histopathological WSIs was compared. After fine-tuning, AlexNet, ResNet, DenseNet, VGG, and Inception models presented better performance than training-from-scratch and pre-trained approaches. The CNNs displayed accuracy rates of 89.42%, 95.25%, 96.98%, 95.86%, and 92.43%, respectively, with fine tuning and enabled rapid scanning and updating of the parameters to cope with the dataset [68]. In another study, the training-from-scratch approach performed better and had the most consistent detection rate across all evaluation criteria, achieving a specificity rate that was 16.81% higher than the best-performing CNN model. The model also produced a detection accuracy of 94.5%, which was 3.85% higher than the highest accuracy rate achieved by other CNN models [74]. Another study evaluated the reliability of the X-CFCMC XAI model by running acceptability tests against a plain CNN model, using feedback from pathologists, and found that the former was more acceptable to pathologists due to its explaining capacity [43].

## 5. Conclusions

Overall, this study concluded that AI is demonstrating promising results in terms of accuracy in the diagnosis of colorectal cancer. However, the user-dependent and complex, non-transparent deep network models do not provide an appropriate level of evidence for the key points used in classification, which is the reason for the slow application of this technique in clinical practice. Most AI models for predicting invasive cancer are prone to over-detection. This suggests that supporting evidence for results of AI-based diagnosis of colorectal cancer is strongly required to continue to optimize model performance for practice-level validation. Therefore, we propose that AI using a visualization method for classification outcomes could significantly reduce the burden on clinicians and improve the diagnostic accuracy for colorectal cancer.

## Figures and Tables

**Table 1:** General summary of studies related to explainable artificial intelligence and sampling methods using deep learning techniques for colon cancer detection.

References	Methods	Imaging Modality
Yao et al., 2020 [80]	Deep Attention Multiple Instance Survival Learning (DeepAttnMISL) with Multiple Instance Fully Convolutional Network (MI-FCN), DeepMISL, Finetuned-WSISA-LassoCOx, Finetuned-WSISA-MTLSA, WSISA-LassoCox, WSISA-MTLSA	Sample images of tissues taken through colonoscopy and turned into WSI clusters.
Sirinukunwattana et al., 2016 [21]	Spatially Constrained Convolutional Neural Network (SC-CNN)	100 H&E-stained histology images of colorectal adenocarcinomas of 500 × 500 pixels were cropped from WSIs using Omnyx VLI20 scanner.
Sabol et al., 2020 [43]	Cumulative Fuzzy Class Membership Criterion (CFCMC)	H&E tissue slides cut into 5000 small sections of 150 × 150 pixels and annotation as one of eight tissue classes.
Korbar et al., 2017 [42]	ResNet	176 H&E-stained WSIs collected from patients who underwent colorectal cancer screening.
Hägele et al., 2020 [76]	GoogLeNet from Caffe Model Zoo	H&E-stained images from TCGA Research Network were chosen and its WSIs were annotated.
Koziarski et al., 2020 [81]	MobileNet	Colorectal cancer histology image datasets divided into 5000 different types of tissues, each with a 150 × 150 pixels dimensionality.
Kainz et al., 2017 [33]	Object-Net and Separator-Net	165 annotated, H&E-stained images of colorectal adenocarcinomas were collected at 20× magnification using a Zeiss MIRAX MIDI Scanner.
Hong et al., 2020 [45]	U-Net with EfficientNet B4 and EfficientNet B5 encoder	Datasets obtained from ETIS-Larib from the MICCAI 2015 polyp detection challenge, and CVCColonDB
Shapcott et al., 2019 [44]	Matconvnet, Tensorflow “cifar10” with Pycharm IDE	142 H&E-stained, 40× magnification colorectal cancer images obtained from TCGA COAD data set from the Genomic Data Commons Portal 2018.

## References

[1] Ferlay J., Soerjomataram I., Siegel R.L., Torre L.A., Jemal A. (2018). Global cancer statistics 2018: GLOBOCAN estimates of incidence and mortality worldwide for 36 cancers in 185 countries. CA Cancer J. Clin..

[2] Wong M.C.S., Huang J., Lok V., Wang J., Fung F., Ding H., Zheng Z.J. (2021). Differences in Incidence and Mortality Trends of Colorectal Cancer Worldwide Based on Sex, Age, and Anatomic Location. Clin. Gastroenterol. Hepatol..

[3] Kahi C.J. (2019). Reviewing the Evidence that Polypectomy Prevents Cancer. Gastrointest. Endosc. Clin. N. Am..

[4] Leggett B., Whitehall V. (2010). Role of the serrated pathway in colorectal cancer pathogenesis. Gastroenterology.

[5] Bejnordi B.E., Zuidhof G., Balkenhol M., Hermsen M., Bult P., van Ginneken B., Karssemeijer N., Litjens G., van der Laak J. (2017). Context-Aware stacked convolutionalneural networks for classification of breast carcinomas in whole-slide histopathology images. J. Med. Imaging.

[6] Gschwantler M., Kriwanek S., Langner E., Goritzer B., SchrutkaKolbl C., Brownstone E., Feichtinger H., Weiss W. (2002). High-grade dysplasia and invasive carcinoma in colorectal adenomas: A multivariate analysis of the impact of adenoma and patient characteristics. Eur. J. Gastroenterol. Hepatol..

[7] Lieberman D.A., Rex D.K., Winawer S.J., Giardiello F.M., Johnson D.A., Levin T.R. (2012). Guidelines for colonoscopy surveillance after screening and polypectomy: A consensus update by the US Multi-Society Task Force on Colorectal Cancer. Gastroenterology.

[8] Vu H.T., Lopez R., Bennett A., Burke C.A. (2011). Individuals with sessile serrated polyps express an aggressive colorectal phenotype. Dis. Colon Rectum.

[9] Miller F.G., Joffe S. (2011). Equipoise and the dilemma of randomized clinical trials. N. Engl. J. Med..

[10] Abdeljawad K., Vemulapalli K.C., Kahi C.J., Cummings O.W., Snover D.C., Rex D.K. (2015). Sessile serrated polyp prevalence determined by a colonoscopist with a high lesion detection rate and an experienced pathologist. Gastrointest. Endosc..

[11] Irshad H., Veillard A., Roux L., Racoceanu D. (2014). Methods for nuclei detection, segmentation, and classification in digital histopathology: A review current status and future potential. IEEE Rev. Biomed. Eng..

[12] Veta M., van Diest P.J., Willems S.M., Wang H., Madabhushi A., Cruz-Roa A., Gonzalez F., Larsen A.B.L., Vestergaard J.S., Dahl A.B. (2015). Assessment of algorithms for mitosis detection in breast cancer histopathology images. Med. Image Anal..

[13] Komura D., Ishikawa S. (2018). Machine learning methods for histopathological image analysis. Comput. Struct. Biotechnol. J..

[14] Malhi A., Kampik T., Pannu H., Madhikermi M., Främling K. (2019). Explaining machine learning-based classifications of in-vivo gastral images. Proceedings of the 2019 Digital Image Computing: Techniques and Applications.

[15] Silva J., Histace A., Romain O., Dray X., Granado B. (2014). Toward embedded detection of polyps in wce images for early diagnosis of colorectal cancer. Int. J. Comput. Assist. Radiol. Surg..

[16] West N.P., Dattani M., McShane P., Hutchins G., Grabsch J., Mueller W., Treanor D., Quirke P., Grabsch H. (2010). The proportion of tumour cells is an independent predictorfor survival in colorectal cancer patients. Br. J. Cancer.

[17] Rastogi A., Keighley J., Singh V., Callahan P., Bansal A., Wani S., Sharma P. (2009). High accuracy of narrow band imaging without magnification for the real-time characterization of polyp histology and its comparison with high-definition white light colonoscopy: A prospective study. Am. J. Gastroenterol..

[18] Lu F.I., de van Niekerk W., Owen D., Turbin D.A., Webber D.L. (2010). Longitudinal outcome study of sessile serrated adenomas of the colorectum: An increased risk for subsequent right-sided colorectal carcinoma. Am. J. Surg. Pathol..

[19] Tischendorf J.J., Gross S., Winograd R., Hecker H., Auer R., Behrens A., Trautwein C., Aach T., Stehle T. (2010). Computer-aided classification of colorectal polyps based on vascular patterns: A pilot study. Endoscopy.

[20] Kitajima K., Fujimori T., Fujii S., Takeda J., Ohkura Y., Kawamata H., Kumamoto T., Ishiguro S., Kato Y., Shimoda T. (2004). Correlations between lymph node metastasis and depth of submucosal invasion in submucosal invasive colorectal carcinoma: A Japanese collaborative study. J. Gastroenterol..

[21] Sirinukunwattana K., Raza S.E.A., Tsang Y.W., Snead D.R., Cree I.A., Rajpoot N.M. (2016). Locality sensitive deep learning for detection and classification of nuclei in routine colon cancer histology images. IEEE Trans. Med. Imaging.

[22] Mobadersany P., Yousefi S., Amgad M., Gutman D.A., Barnholtz-Sloan J.S., Velázquez Vega J.E., Brat D.J., Cooper L.A. (2018). Predicting cancer outcomes from histology and genomics using convolutional networks. Proc. Natl. Acad. Sci. USA.

[23] Itoh T., Kawahira H., Nakashima H., Yata N. (2018). Deep learning analyzes *Helicobacter pylori* infection by upper gastrointestinal endoscopyimages. Endosc. Int. Open.

[24] Korbar B., Olofson A.M., Miraflor A.P., Nicka C.M., Suriawinata M.A., Torresani L., Suriawinata A.A., Hassanpour S. (2017). Deep learning for classification of colorectal polyps on whole-slide images. J Pathol. Inform..

[25] Komeda Y., Handa H., Watanabe T., Nomura T., Kitahashi M., Sakurai T., Okamoto A., Minami T., Kono M., Arizumi T. (2017). Computeraided diagnosis based on convolutional neural network system for colorectal polyp classification: Preliminary experience. Oncology.

[26] Wimmer G., Tamaki T., Tischendorf J.J., Häfner M., Yoshida S., Tanaka S., Uhl A. (2016). Directional wavelet based features for colonic polyp classification. Med. Image Anal..

[27] Hafner M., Tamaki T., Tanaka S., Uhl A., Wimmer G., Yoshida S. (2015). Local fractal dimension based approaches for colonic polyp classification. Med. Image Anal..

[28] Okamoto T., Koide T., Sugi K., Shimizu T., Hoang A.T., Tamaki T., Raytchev B., Kaneda K., Kominami Y., Yoshida S. Image segmentation of pyramid style identifier based on Support Vector Machine for colorectal endoscopic images. Proceedings of the 2015 37th Annual International Conference of the IEEE Engineering in Medicine and Biology Society (EMBC).

[29] Mori Y., Kudo S.E., Chiu P.W., Misawa M., Wakamura K., Kudo T., Hayashi T., Katagiri A., Miyachi H., Ishida F. (2016). Impact of an automated system for endocytoscopic diagnosis of small colorectal lesions: An international web-based study. Endoscopy.

[30] Ştefănescu D., Streba C., Cârţână E.T., Săftoiu A., Gruionu G., Gruionu L.G. (2016). Computer Aided Diagnosis for Confocal Laser Endomicroscopy in Advanced Colorectal Adenocarcinoma. PLoS ONE.

[31] Trebeschi S., van Griethuysen J.J.M., Lambregts D.M.J., Parmar C., Bakers F.C., Peters N.H., Beets-Tan R.G., Aerts H.J. (2017). Deep Learning for Fully-Automated Localization and Segmentation of Rectal Cancer on Multiparametric MR. Sci. Rep..

[32] Zhao X.P., Xie P., Wang M., Li W., Pickhardt P.J., Xia W., Xiong F., Zhang R., Xie Y., Jian J. (2020). Deep learning-based fully automated detection and segmentation of lymph nodes on multiparametric-mri for rectal cancer: A multicentre study. EBioMedicine.

[33] Kainz K., Pfeiffer M., Urschler M. (2017). Segmentation and classification of colon glands with deep convolutional neural networks and total variation regularization. PeerJ.

[34] Urban G., Tripathi P., Alkayali T., Mittal M., Jalali F., Karnes W., Baldi P. (2018). Deep learning localizes and identifies polyps in real time with 96% accuracy in screening colonoscopy. Gastroenterology.

[35] Tajbakhsh N., Gurudu S.R., Liang J. (2016). Automated polyp detection in colonoscopy videos using shape and context information. IEEE Trans. Med. Imaging.

[36] Misawa M., Kudo S.E., Mori Y., Cho T., Kataoka S., Yamauchi A., Ogawa Y., Maeda Y., Takeda K., Ichimasa K. (2018). Artificial intelligence assisted polyp detection for colonoscopy: Initial experience. Gastroenterology.

[37] Wang P., Xiao X., Glissen Brown J.R., Berzin T.M., Tu M., Xiong F., Hu X., Liu P., Song Y., Zhang D. (2018). Development and validation of a deep-learning algorithm for the detection of polyps during colonoscopy. Nat. Biomed. Eng..

[38] Ding H., Pan Z., Cen Q., Li Y., Chen S. (2020). Multi-scale fully convolutional network for gland segmentation using three-class classification. Neurocomputing.

[39] Yoon H., Lee J., Oh J.E., Kim H.R., Lee S., Chang H.J., Sohn D.K. (2019). Tumor Identification in Colorectal Histology Images Using a Convolutional Neural Network. J. Digit. Imaging.

[40] Sena P., Fioresi R., Faglioni F., Losi L., Faglioni G., Roncucci L. (2019). Deep learning techniques for detecting preneoplastic and neoplastic lesions in human colorectal histological images. Oncol. Lett..

[41] Xu Y., Jia Z., Wang L.B., Ai Y., Zhang F., Lai M., Chang E.I. (2017). Large scale tissue histopathology image classification, segmentation, and visualization via deep convolutional activation features. BMC Bioinform..

[42] Korbar B., Olofson A.M., Miraflor A.P., Nicka C.M., Suriawinata M.A., Torresani L., Suriawinata A.A., Hassanpour S. Looking under the hood: Deep neural network visualization to interpret whole-slide Image analysis outcomes for colorectal polyps. Proceedings of the IEEE Conference on Computer Vision and Pattern Recognition Workshops.

[43] Sabol P., Sinčák P., Hartono P., Kočan P., Benetinová Z., Blichárová A., Verbóová Ľ., Štammová E., Sabolová-Fabianová A., Jašková A. (2020). Explainable classifier for improving the accountability in decision-making for colorectal cancer diagnosis from histopathological images. J. Biomed. Inform..

[44] Mary S., Hewitt K.J., Rajpoot N. (2019). Deep Learning with Sampling in Colon Cancer Histology. Front. Bioeng. Biotechnol..

[45] Hong L.T.T., Thanh N.C., Long T.Q. Polyp segmentation in colonoscopy Images using ensembles of U-Nets with efficientNet and asymmetric similarity loss function. Proceedings of the 2020 International Conference on Computing and Communication Technologies (RIVF).

[46] Yamada M., Saito Y., Imaoka H., Saiko M., Yamada S., Kondo H., Takamaru H., Sakamoto T., Sese J., Kuchiba A. (2019). Development of a real-time endoscopic image diagnosis support system using deep learning technology in colonoscopy. Sci. Rep..

[47] Ho C., Zhao Z., Chen X.F., Sauer J., Saraf S.A., Jialdasani R., Taghipour K., Sathe A., Khor L.-Y., Lim K.-H. (2022). A promising deep learning assistive algorithm for histopathological screening of colorectal cancer. Sci. Rep..

[48] Xu L., Walker B., Liang P.-I., Tong Y., Xu C., Su Y.C., Karsan A. (2020). Colorectal cancer detection based on deep learning. J. Pathol. Inform..

[49] Buendgens L., Didem C., Laleh N.G., van Treeck M., Koenen M.T., Zimmermann H.W., Herbold T., Lux T.J., Hann A., Trautwein C. (2022). Weakly supervised end-to-end artificial intelligence in gastrointestinal endoscopy. Sci. Rep..

[50] Takamatsu M., Yamamoto N., Kawachi H., Chino A., Saito S., Ueno M., Ishikawa Y., Takaawa Y., Takeuchi K. (2019). Prediction of early colorectal cancer metastasis by machine learning using digital slide images. Comput. Methods Programs Biomed..

[51] Ghosh J., Sharma A.K., Tomar S. (2021). Feature extraction and classification of colon cancer using a hybrid approach of supervised and unsupervised learning. Advanced Machine Learning Approaches in Cancer Prognosis.

[52] Thomaz V.A., Sierra-Franco C.A., Raposo A.B. (2020). Training data enhancements for improving colonic polype detection using deep convolutional neural networks. Aritificial Intell. Med..

[53] Yu G., Sun K., Xu C., Shi X.-H., Wu C., Xie T., Meng R.-Q., Meng X.-H., Wang K.-S., Xiao H.-M. (2021). Accurate recognition of colorectal cancer with semi-supervised deep learning on pathological images. Nat. Commun..

[54] Rączkowski L., Mozejko M., Zambonelli J., Szczurek E. (2019). Ara: Accurate, reliable and active histopathological image classification framework with Bayesian deep learning. Sci. Rep..

[55] Mori Y., Kudo S.-E., Wakamura K., Misawa M., Ogawa Y., Kutsukawa M., Kudo T., Hayashi T., Miyachi H., Ishida F. (2015). Novel computer-aided diagnostic system for colorectal lesions using endoscytoscopy. Gastrointest. Endosc..

[56] Nartowt B.J., Hart G.R., Muhammad W., Liang Y., Stark G.F., Deng J. (2020). Robust Machine Learning for Colorectal Cancer Risk Prediction and Stratification. Front. Big Data.

[57] Wan J.-J., Chen B.-L., Kong Y.-X., Ma X.-G., Yu Y.-T. (2019). An early intestinal cancer prediction algorithm based on deep belief network. Sci. Rep..

[58] Ito I., Kawahira H., Nakashima H., Uesato M., Miyauchi H., Matsubara H. (2019). Endoscopic diagnostic support system for cT1b colorectal cancer using deep learning. Oncology.

[59] Tamai N., Saito Y., Sakamoto T., Nakajima T., Matsuda T., Sumiyama K., Tajiri H., Koyama R., Kido S. (2017). Effectiveness of computer-aided diagnosis of colorectal lesions using novel software for magnifying narrow-band imaging: A pilot study. Endosc. Int. Open.

[60] Song Z., Yu C., Zou S., Wang W., Huang Y., Ding X., Liu J., Shao L., Yuan J., Gou X. (2020). Automatic deep learning-based colorectal adenoma detection system and its similarities with pathologists. BMJ Open.

[61] Rathore S., Hussain M., Khan A. (2015). Automated colon cancer detection using hybrid of novel geometric features and some traditional features. Comput. Biol. Med..

[62] Nadimi E.S., Buijs M.M., Herp J., Kroijer R., Kobaek-Larsen M., Nielsen E., Pedersen C.D., Blanes-Vidal V., Baatrup G. (2020). Application of deep learning for autonomous detection and localization of colorectal polyps in wireless colon capsule endoscopy. Comput. Electr. Eng..

[63] Glasmachers T. (2017). Limits of end-to-end learning. Proc. Mach. Learn. Res..

[64] Graves A., Wayne G., Danihelka I. (2014). Neural turing machines. arXiv.

[65] Graves G., Wayne M., Reynolds T., Harley I., Danihelka A., Grabska-Barwi’nska S., Colmenarejo G’omez E., Grefenstette T.R., Agapiou J. (2016). Hybrid computing using a neural network with dynamic external memory. Nature.

[66] Tamar A., Levine S., Abbeel P., Wu Y., Thomas G. (2016). Value iteration networks. Advances in Neural Information Processing Systems, Proceedings of the 30th Conference on Neural Information Processing Systems (NIPS 2016), Barcelona, Spain, 5–10 December 2016.

[67] Mirowski P., Pascanu R., Viola F., Soyer H., Ballard A., Banino A., Denil M., Goroshin R., Sifre L., Kavukcuoglu K. (2016). Learning to navigate in complex environments. arXiv.

[68] Iizuka O., Kanavati F., Kato K., Rambeau M., Arihiro K., Tsuneki M. (2020). Deep learning models for histopathological classification of gastric and colonic epithelial tumours. Sci. Rep..

[69] Pinckaers H., Litjens G. (2019). Neural ordinary differential equation for semantic segmentation of individual colon glands. arXiv.

[70] Olivas E.S., Guerrero J.D.M., Sober M.M., Benedito J.R.M., Lopez A.J.S. (2009). Handbook of Research on Machine Learning Applications and Trends: Algorithms, Methods and Techniques.

[71] Dilmegani C. Transfer Learning in 2022: What It Is & How It Works. Artificial Intelligence Multiple, 2020. https://research.aimultiple.com/transfer-learning/.

[72] Gessert N., Bengs M., Wittig L., Drömann D., Keck T., Schlaefer A., Ellebrecht D.B. (2019). Deep transfer learning methods for colon cancer classification in confocal laser microscopy images. Int. J. Comput. Assist. Radiol. Surg..

[73] Hamida A.B., Devanne M., Weber J., Truntzer C., Derangère V., Ghiringhelli F., Forestier G., Wemmert C. (2021). Deep learning for colon cancer histopathological images analysis. Computers in Biology and Medicine.

[74] Malik J., Kiranyaz S., Kunhoth S., Ince T., Al-Maadeed S., Hamila R., Gabbouj M. (2019). Colorectal cancer diagnosis from histology images: A comparative study. arXiv.

[75] Kather J.N., Krisam J., Charoentong P., Luedde T., Herpel E., Weis C.-A., Gaiser T., Marx A., Valous N.A., Ferber D. (2019). Predicting survival from colorectal cancer histology slides using deep learning: A retrospective multicentre study. PLoS Med..

[76] Hägele M., Seegerer P., Lapuschkin S., Bockmayr M., Samek W., Klauschen F., Mülle K.-R., Binder A. (2020). Resolving challenges in deep learning-based analyses of histopathological images using explanation methods. Sci. Rep..

[77] Kather J.N., Pearson A.T., Halama N., Jäger D., Krause J., Loosen S.H., Marx A., Boor P., Tacke F., Neumann U.P. (2019). Deep learning can predict microsatellite instability directly from histology in gastrointestinal cancer. Nat. Med..

[78] Bychkov D., Linder N., Turkki R., Nordling S., Kovanen P.E., Verrill C., Walliander M., Lundin M., Haglund C., Lundin J. (2018). Deep learning based tissue analysis predicts outcome in colorectal cancer. Sci. Rep..

[79] Ponzio F., Macii E., Ficarra E., Di Cataldo S. Colorectal cancer classification using deep convolutional networks. Proceedings of the 11th International Joint Conference on Biomedical Engineering Systems and Technologies 2018.

[80] Yao J., Zhu X., Jonnagaddala J., Hawkins N., Huang J. (2020). Whole slide images based cancer survival prediction using attention guided deep multiple instance learning networks. Med. Image Anal..

[81] Koziarski M. (2020). Two-Stage Resampling for Convolutional Neural Network Training in the Imbalanced Colorectal Cancer Image Classification. arXiv.

[82] Li Y., Eresen A., Shangguan J., Yang J., Lu Y., Chen D., Wang J., Velichko Y., Yaghmai V., Zhang Z. (2019). Establishment of a new non-invasive imaging prediction model for liver metastasis in colon cancer. Am. J. Cancer Res..

[83] Wang K.S., Yu G., Xu C., Meng X.H., Zhou J., Zheng C., Deng Z., Shang L., Liu R., Su S. (2021). Accurate diagnosis of colorectal cancer based on histopathology images using artificial intelligence. BMC Med..

